# Sensory Ganglia-Specific TNF Expression Is Associated With Persistent Nociception After Resolution of Inflammation

**DOI:** 10.3389/fimmu.2019.03120

**Published:** 2020-01-20

**Authors:** William Antonio Gonçalves, Barbara Maximino Rezende, Marcos Paulo Esteves de Oliveira, Lucas Secchim Ribeiro, Victor Fattori, Walison Nunes da Silva, Pedro Henrique Dias Moura Prazeres, Celso Martins Queiroz-Junior, Karina Talita de Oliveira Santana, Walyson Coelho Costa, Vinícius Amorim Beltrami, Vivian Vasconcelos Costa, Alexander Birbrair, Waldiceu A. Verri, Fernando Lopes, Thiago Mattar Cunha, Mauro Martins Teixeira, Flávio Almeida Amaral, Vanessa Pinho

**Affiliations:** ^1^Departamento de Morfologia, Instituto de Ciências Biológicas (ICB), Universidade Federal de Minas Gerais (UFMG), Belo Horizonte, Brazil; ^2^Departamento de Enfermagem Básica, Escola de Enfermagem da Universidade Federal de Minas Gerais (UFMG), Belo Horizonte, Brazil; ^3^Biomediziniches Zentrum (BMZ), Institut für Angeborene Immunität, Rheinische Friedrich-Wilhelms-Universität Bonn, Venusberg, Germany; ^4^Departamento de Patologia, Center of Biological Sciences, Londrina State University, Londrina, Brazil; ^5^Departamento de Patologia, Instituto de Ciências Biológicas (ICB), Universidade Federal de Minas Gerais (UFMG), Belo Horizonte, Brazil; ^6^Departamento de Genética, Ecologia e Evolução, Instituto de Ciências Biológicas (ICB), Universidade Federal de Minas Gerais (UFMG), Belo Horizonte, Brazil; ^7^Institute of Parasitology and Department of Microbiology and Immunology, McGill University, Montreal, QC, Canada; ^8^Departamento de Farmacologia, Faculdade de Medicina de Ribeirão Preto, Universidade de São Paulo (USP), Ribeirão Preto, Brazil; ^9^Departamento de Bioquímica e Imunologia, Instituto de Ciências Biológicas (ICB), Universidade Federal de Minas Gerais (UFMG), Belo Horizonte, Brazil

**Keywords:** resolution of inflammation, arthritis, pain, TNF, neuroinflammation, dorsal root ganglia

## Abstract

Joint pain is a distressing symptom of arthritis, and it is frequently persistent even after treatments which reduce local inflammation. Continuous production of algogenic factors activate/sensitize nociceptors in the joint structures and contribute to persistent pain, a challenging and difficult condition to treat. TNF is a crucial cytokine for the pathogenesis of several rheumatic diseases, and its inhibition is a mainstay of treatment to control joint symptoms, including pain. Here, we sought to investigate the inflammatory changes and the role of TNF in dorsal root ganglia (DRG) during persistent hypernociception after the resolution of acute joint inflammation. Using a model of antigen-induced arthritis, the peak of joint inflammation occurred 12–24 h after local antigen injection and was characterized by an intense influx of neutrophils, pro-inflammatory cytokine production, and joint damage. We found that inflammatory parameters in the joint returned to basal levels between 6 and 8 days after antigen-challenge, characterizing the resolving phase of joint inflammation. Mechanical hyperalgesia was persistent up to 14 days after joint insult. The persistent nociception was associated with the inflammatory status of DRG after cessation of acute joint inflammation. The late state of neuroinflammation in the ipsilateral side was evidenced by gene expression of TNF, TNFR2, IL-6, IL-1β, CXCL2, COX2, and iNOS in lumbar DRG (L3-L5) and leukocyte adhesion in the lumbar intumescent vessels between days 6 and 8. Moreover, there were signs of resident macrophage activation in DRG, as evidenced by an increase in Iba1-positive cells. Intrathecal or systemic injection of etanercept, an agent clinically utilized for TNF neutralization, at day 7 post arthritis induction, alleviated the persistent joint hyperalgesia by specific action in DRG. Our data suggest that neuroinflammation in DRG after the resolution of acute joint inflammation drives continuous neural sensitization resulting in persistent joint nociception in a TNF-dependent mechanism.

## Introduction

The inflammatory process, a protective response against various infectious and sterile noxious stimuli, is responsible for the elimination of the stressor agent resulting in a return to tissue homeostasis (1, 2). However, continuous inflammation is associated with persistent harmful stimuli or failed inflammatory resolution (3–5). The resolution of inflammation is a well-controlled process resulting in a reduction of leukocyte accumulation and an increase in neutrophil apoptosis, macrophages reprogramming, and functional recovery of tissue. In joint inflammation, pain is an important cardinal signal associated with loss of function. Previous studies have demonstrated that articular hyperalgesia may persist after resolution of inflammation in a model of antigen induced-arthritis (6, 7). The mechanism which coordinates this persistent articular pain following inflammatory cessation is unknown.

Joint pain is initiated by the activation of specialized neuronal fibers called nociceptors, these include afferent C and Aδ fibers (8–11). These peripheral sensory nerve terminals in the joints detect a plethora of algogenic molecules during inflammation, including several immune mediators, such as cytokines released by activated resident and migrated cells, in different contexts of arthritis (12–14). In this context, the elicited response to nociceptive stimuli is exacerbated (15) resulting in an increased intensity and frequency of transduced action potentials from the affected joint neurons to the central nervous system, which modulate the pain pathways (16). Patients affected by arthritis can experience persistent pain in the joint without any obvious signs of inflammatory activity (17–20). The response in the central nervous system is one of the most studied mechanisms underlying this phenomenon, and both spinal cord neuroinflammation and microglial cell activation drive hyperresponsivity of nociceptive pathways (16, 21–23).

TNF is a crucial cytokine for the development of articular pain during arthritis (12, 24). The neutralization of TNF by anti-TNF drugs is an important pharmacological strategy for decreasing disease activity and joint pain (25, 26). Experimentally, the neutralization of spinal TNF by intrathecal treatment with infliximab, an anti-TNF monoclonal antibody, decreased joint nociception in a mouse model of antigen-induced arthritis (AIA) (27). Moreover, the intraarticular injection of etanercept, a fusion protein based on the TNF receptor type 2 (TNFR2), reduces the responsivity of articular nociceptors to mechanical stimulation. This suggests that TNF acts in peripheral neuronal terminals during inflammatory phases in AIA (28, 29). However, despite central and peripheral mechanisms producing articular pain, the role of TNF in driving persistent joint pain in the dorsal root ganglia (DRG) after resolution of joint inflammation is still poorly understood.

The hyperalgesia observed during active joint inflammation is associated with the actions of TNF in the peripheral sites (6) and hyperexpression of its receptors, TNFR1 and TNFR2, in the DRG (30, 31). Furthermore, nociceptive responses decrease following TNF neutralization by systemic injection of anti-TNF drug (6) or etanercept (30, 31). However, the specific site where this cytokine was neutralized, leading to pain relief has, until now, remained unknown. In addition, the role of TNF in maintaining pain during the late stages of an inflammatory response when the productive phase of cell recruitment and cytokine production have subsided remains unknown. Therefore, in this work we aimed to investigate the role of TNF expressed in the DRG in driving persistent joint nociception even during the resolving phase of joint inflammation in an experimental model of arthritis in mice.

## Methods

### Animals

Seven to twelve-week-old male age-matched C57Bl/6 mice (20 to 25 g) were used in animal experiments. These animals were housed in ventilated micro isolator cages in a temperature-controlled room (22 to 25°C), under a 12 h light/dark cycle with *ad libitum* access to water and food. All procedures were approved by the animal ethics committee of the Federal University of Minas Gerais (51/2018).

### Antigen-Induced Arthritis

The immunization procedure was performed as previously described (7, 32). Anesthetised (100 μl of a mixture of 100 mg/kg of ketamine and 15 mg/kg of xylazine, intraperitoneally) mice were sensitized by intradermal (i.d.) injection of 500 μg of methylated bovine serum albumin (mBSA) dissolved in an emulsion containing 50 μl of phosphate buffer solution (PBS) and 50 μl of complete Freund's adjuvant (CFA; 1 mg/mL of *Mycobacterium tuberculosis*). For AIA induction, 14 days after emulsion injection, the right knees of mice were challenged with an intra-articular injection solution containing 10 μg of mBSA dissolved in 10 μl of sterile PBS.

### Analysis of Cell Accumulation in the Joint

Groups of mice were culled with an overdose of anesthesia (100 μl of a mixture of 180 mg/kg of ketamine and 24 mg/kg of xylazine, intraperitoneally) and the knee joint cavity was surgically exposed and washed with 10 μL of PBS contained 3% (w/v) of bovine serum albumin. The total number of leukocytes were determined using a Neubauer chamber and Turk's solution. Neutrophil and mononuclear cell counts were performed using Cytospin (Shandon III) preparations by evaluating the percentage of each leukocyte type on a slide stained with Panoptic solutions (Laborclin, PR, Brazil) and subsequently differentiated by light microscopy for quantification.

### Histology

Tibiofemoral joint samples were collected for histopathological evaluation. The samples were fixed in 10% (v/v) buffered formalin (pH 7.4), decalcified in 14% EDTA (w/v) for 4 weeks, embedded in paraffin, sectioned, placed in slides, and stained with H&E. The H&E sections were examined and scored by a pathologist in a blinded manner for the following parameters: inflammatory infiltrate intensity, severity of synovial membrane hyperplasia, presence of inflammatory cells in the synovial space, and bone resorption. The grades were summed to obtain a histologic score (ranging from 0 to 12) as previously described (33).

### Intravital Microscopy

After AIA induction, synovial (34) or spinal cord venules were exposed for the quantification of rolling and adhesion leukocytes by intravital microscopy. For knee joint analysis, the quadriceps tendon was carefully sectioned and rebounded to the opening target area presenting venules underlying the synovial tissue. For the spinal cord analysis, we used a laminectomy of vertebrates that involved lumbar intumescence exposing dorsal microvasculature of the dorsal horn of the spinal cord. Following the surgery, the knee was slightly flexed, or mice were correctly positioned allowing examination of articular or spinal microcirculation, respectively, using a 20-fold objective from intravital microscope (ECLIPSE 50i; Nikon). A digital camera (DS-Qi1MC; Nikon) was used to capture images. Registration of experiment were performed using Nikon imaging software. Rolling leukocytes were defined as cells that moved at a velocity less than that of the erythrocytes within a given vessel. The rolling cell flux was measured in venules with 20–40 μm, determined as the number of rolling cells that passed by a given point per minute. Leukocytes were considered to be adherent if they remained stationary for ≥30 s, and total leukocyte adhesion was quantified as the number of adherent cells in the intravascular space within an area of 100 μm.

### Hyperalgesia Articular Evaluation

For environmental adaptation, mice were placed in acrylic cages (12 x 10 x 17 cm in height) with a wire-grid floor, in a noised controlled room, for 60 min. After this time, exploratory behavior manifestation was abrogated, and all mice remained quiet allowing for nociceptive response evaluation. To identify the withdrawal threshold, we used an electronic von Frey algesimeter (INSIGHT Instruments, Ribeirão Preto, SP, Brazil). Using a hand-held force transducer, fitted with a polypropylene tip (4.15 mm), the observer applied a vertical and constant force in central plantar surface of mice paw. This procedure was intended to produce an articular mechanical stimulus for knee flexion. The sufficient force in grams (g) to trigger a paw withdrawal movement, the characteristic aversive behavior to avoid the incident stress, was recorded by an electronic component of the apparatus. The withdrawal threshold was calculated by replicating the procedure in triplicate for each mouse (and averages were expressed in absolute values). Basal response was measured 24 h before saline or mBSA injections. After AIA induction, the nociceptive response was measured within 24 h, and subsequent time points with 48 h of interval.

### Intrathecal Injection

Intrathecal injection was performed in intervertebral space between the fifth and sixth lumbar vertebrates (L5/L6). A volume of 5 μL was injected with a 30G needle in anesthetised animals. The injection was considered correct when mice presented a tail reflex after needle insertion (35).

### Gene Expression by qRT-PCR

DRG and spinal cords were collected for mRNA expression analyses. Briefly, mice were anesthetised and culled at different time points after AIA induction or PBS challenge. Subsequently, vertebrae laminectomy was performed to allow removal of the lumbar DRG (L3-L5) and its respective spinal cord segments. These segments were separated in ipsi- and contralateral portions, or only the dorsal horn section of the ipsilateral portion. We used Trizol reagent (Invitrogen Life Technologies Corporation- Carlsbad, CA, USA) for total RNA extraction from collected tissues according to the manufacturer's instructions. Total RNA purity was determined using a Nanodrop 1000 spectrophotometer (Thermo Scientific- Waltham, MA, USA). The wavelength absorption rate (260/230 nm and 280 /260 nm) between 1.8 and 2.0, respectively, was presented for all samples. A mix containing the reserve transcriptase, SuperScript III, ribonuclease recombinant inhibitor (RNAse Out; Invitrogen Life Technologies Corporation- Carlsbad, CA, USA) and dithiothreitol (DTT; 1 mM) were used for reverse transcription of total RNA into cDNA. For quantitative PCR the Power SYBR Master Mix reagent (Invitrogen Life Technologies Corporation- Carlsbad, CA, USA) and initiators pars (Integrated DNA Technologies- Coralville, IA, EUA) plus cDNA were placed into a 96-well plate in duplicate. Next, a 7500 Fast Real-Time PCR System (Applied Biosystems, Waltham, MA, EUA) was used for performing the programmed reaction: initial heating at 95 °C for 10 min, following by 40 cycles at 95°C for 60 seconds and 60°C for 1 min. The 2-ΔΔCT method (36) was used to calculate the cycle threshold (CT), and the fold change was normalized to glyceraldehyde 3-phosphate dehydrogenase (GAPDH) levels and expressed as fold change compared to the PBS-treated controls.

### Western Blot

Lumbar DRG were homogenized in a lysate solution buffer (1% Triton X-100, 100 mM Tris/ HCl, pH 8.0, 10% (v/v) glycerol, 5 mM EDTA, 200 mM NaCl, 1 mM DTT, 1 mM PMSF, 2.5 μg/mL leupeptin, 5 μg/mL aprotinin, 1 mM sodium orthovanadate). Each poll of ganglia contained L4 from three different animals. The lysate protein concentration was quantified with a Bradford assay (Bio-Rad, Hercules, CA, USA). Sodium dodecyl sulfate polyacrylamide gel electrophoresis (SDS-PAGE) was carried with 30 μg of proteins per sample. After separation by SDS-PAGE, proteins were transferred onto a nitrocellulose membrane (Hybond ECL, GE Healthcare). The membrane was then incubated with a goat monoclonal primary antibody against TNF (1:1000, Cat: sc1348, Santa Cruz Biotechnology, Inc) and GAPDH (HRP conjugate; 1:1000, Cat: #3686, Cell Signaling, Inc) over night at 4°C. After incubation with the anti-TNF primary antibody, the membrane was washed 3 times for 5 min each wash with PBS 0.1% Tween-20 before being incubated with a rabbit anti-goat IgG-HRP secondary antibody (1:3000, Cat: sc2768, Santa Cruz Biotechnology, Inc) for 3 h at room temperature. Immunoreactive bands were visualized on photosensitive film after exposure to enhanced chemiluminescence (ECL) solution.

### ELISA

The protein concentration of cytokines from articular tissue, namely the infrapatellar fat pad, meniscus, synovial membrane, joint capsule, patella, patellar tendon, and synovia, was measured at different time points after AIA induction. The collected tissues were mixed in a homogenizer (Quiagen, Biotecnologia Brasil Ltda, São Paulo, SP, Brazil) for 5 min with a solution that contained antiproteases (0.1 mM PMSF), 0.1 nM benzethonium chloride, 10 mM EDTA, 20 Kallikrein inhibitor units, aprotinin A, and 0.05% (v/v) Tween-20. The samples were centrifuged for 10 min at 10,000 rpm at 4°C. The supernatants were analyzed by enzyme-linked immunosorbent assay (ELISA). The concentrations of analyzed cytokines were measured according to the manufacturer's instructions (R&D systems). Colorimetric reactions were analyzed with a spectrophotometer at 492 nm.

### Immunofluorescence

L4 DRGs were dissected, post-fixed, and incubated overnight with 30% (w/v) sucrose. DRGs were embedded in optimum cutting temperature, and 20 μm sections were cut in a cryostat and processed for immunofluorescence. The primary antibodies used in this study were: anti-cfos (1:500, cat #MA1-21190, Thermo Fisher Scientific, Waltham, MA, USA) and anti-Iba 1 (1:100, cat #PA5-21274, Thermo Fisher Scientific, Waltham, MA, USA). Secondary antibodies were: Alexa Fluor 647 (1:500, cat #A32733, Thermo Fisher Scientific, Waltham, MA, USA), Alexa Fluor 488 (1:1000, cat #A11001, Thermo Fisher Scientific, Waltham, MA, USA. DAPI (1:1000, Thermo Fisher Scientific, Waltham, MA, USA) was used as a nucleus marker. The coverslips were fixed on slides with Fluormount (00-4958-02, Thermo Fisher Scientific, Waltham, MA, USA). Imaging was performed using a confocal microscope (Leica TCS SP8, Leica Microsystems, Mannheim, Germany). The fluorescence intensity was quantified using LAS X Software (Leica Microsystems, Mannheim, Germany) and macrophages present in DRG were quantified by counting the total iba1 positive marked cells in the histological sections.

### Statistical Analysis

Graph-Pad Prism version 6 was used for statistical analysis. Data are expressed as the means ± SEM. Comparisons among the groups were performed by unpaired Student's t test or ANOVA, followed by Dunnett *post-hoc* analysis. Two-way ANOVA was adopted to compare the nociceptive response among the groups and/or doses at different times in the curve. The *post-hoc* tests were determined in accordance with recommendation of utilized statistical software. Statistical significance was set as *p* < 0.05.

## Results

### Temporal Characterization of Resolution of Acute Joint Inflammation in the AIA Model

**Figure 1:**
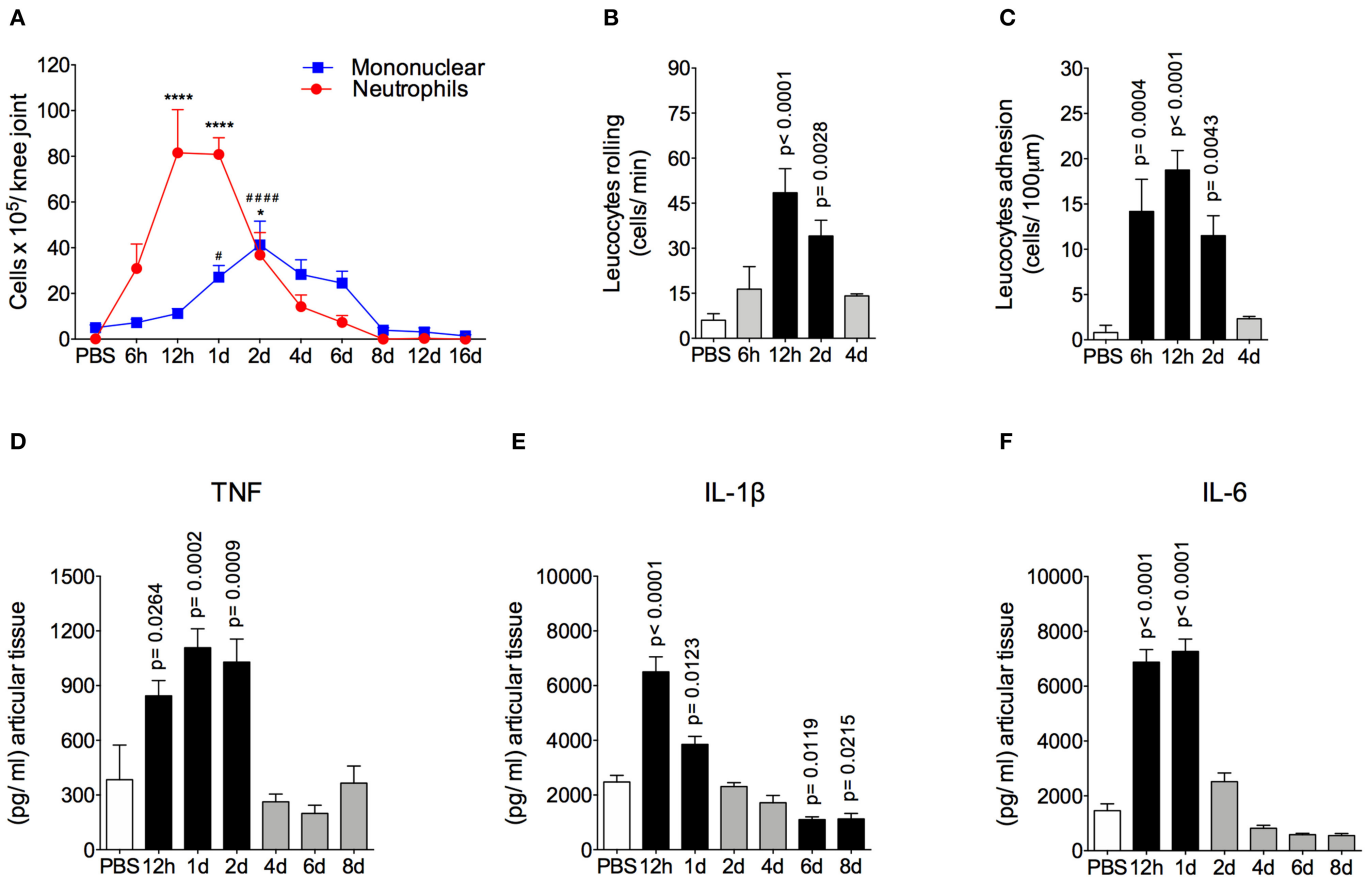
Time course of inflammatory response in AIA model. **(A)** Number of neutrophils and mononuclear cells in the knee joint cavity at different time points after injection of 10 μg of mBSA (10 μg/10 μl of PBS), as well as 12 h after injection of 10 μl of sterile phosphate buffered saline (PBS) into the knee joints of immunized mice (*n* = 4–6). **(B)** Rolling and **(C)** adhesion of leukocytes in knee joint microvasculature (*n* = 5). Concentration of **(D)** TNF, **(E)** IL-1β and **(F)** IL-6 in the joint tissue homogenate were evaluated by ELISA (*n* = 5). The results are represented as mean ± SEM; *p < 0.05, ****(to neutrophils), and ^####^ (to mononuclear cells) *p* < 0.0001 (A) or *p* = specified value in the graph **(B–F)** compared to control group (PBS) using one-way ANOVA following by Dunnett *post-hoc* analysis.

The AIA model is characterized by a rapid and massive accumulation of leukocytes, mostly neutrophils, into the challenged joint (6, 7). The time course and intensity of the inflammatory response in this model is shown in Figure 1. We found that there was a peak of neutrophil accumulation in the articular cavity at 12 h and 1 day after AIA induction. Moreover, mononuclear cells peaked 2 days after intraarticular challenge (Figure 1A). The intravital analysis of the joint microvasculature showed an increase in rolling cells from 12 h after joint insult that was still elevated after up to 2 days (Figure 1B). The increase in adherent cells started from 6 h after AIA induction (Figure 1C). Importantly, 4 days after AIA induction, the number of rolling and adherent cells returned to basal levels.

Leukocyte depuration and pro-inflammatory cytokine clearance are important steps for the efficient resolution of acute joint inflammation and return to tissue homeostasis (37, 38). Here, neutrophils were gradually removed from inflamed cavity between 2 and 6 days after arthritis induction. Mononuclear cells depuration started at a later time point. However, both cells types had been eliminated from the joint at day 8 after AIA induction (Figure 1A), characterizing an effective resolution of acute joint inflammation. The levels of TNF in the articular tissue increased 12 to 48 h after AIA induction, but returned to the control levels within 4 to 8 days (Figure 1D). In addition, levels of IL-1β and IL-6 were increased at 12 h and 1 day after AIA induction and returned to basal levels on day 2 (Figures 1E,D).

**Figure 2:**
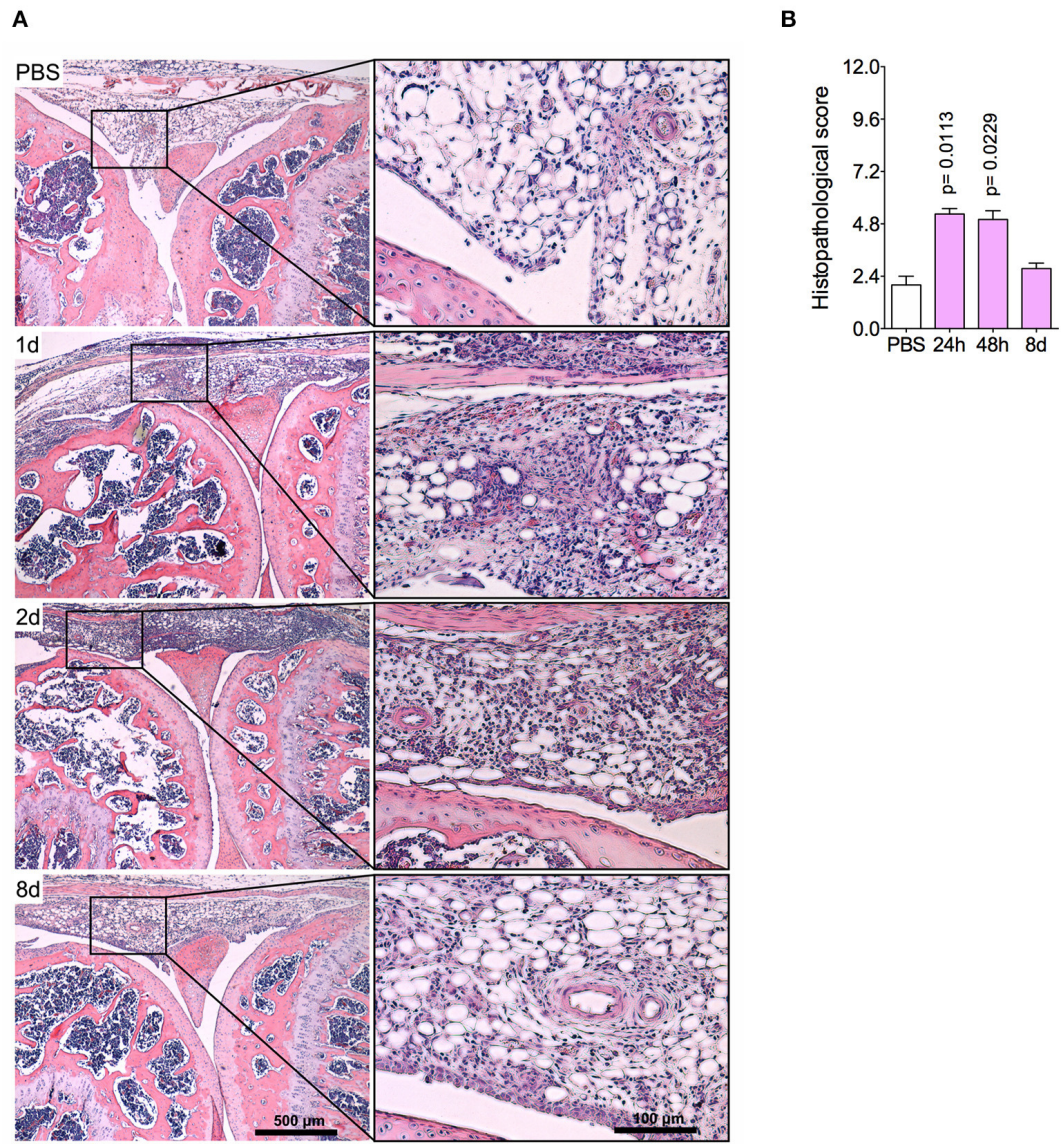
Histopathological analyses of the knee joint in AIA model. Immunized mice received either i.a. injection of mBSA (10 μg/10 μl of PBS) to AIA induction or PBS (10 μl) in control group. **(A)** Representative histological slide to H&E of challenged knee joint. **(B)** Histopathological score quantification of **(A)**. Knee joint samples from the control group were extracted 24 h after i.a. injection (*n* = 4–5). The results are represented as mean ± SEM; *p* = specified value in the graph compared to control group (PBS) using one-way ANOVA following by Dunnett *post-hoc* analysis.

The histopathological analysis corroborated previous findings, indicating an intense leukocyte infiltrate, predominantly of neutrophils, both in the synovial tissue and cavity at 1 day and 2 days post-challenge (Figures 2A,B). Focal hyperplasia of the synovial membrane and scarce lacunae of bone resorption were also detected. Altogether, this set of results evidences a regression of tissue inflammation in this acute model of arthritis, indicating a complete resolution of acute joint inflammation 8 days after arthritis induction.

### Prolonged Joint Nociception During the Resolving Phase of Arthritis Is Associated With an Inflammatory State in DRG

**Figure 3:**
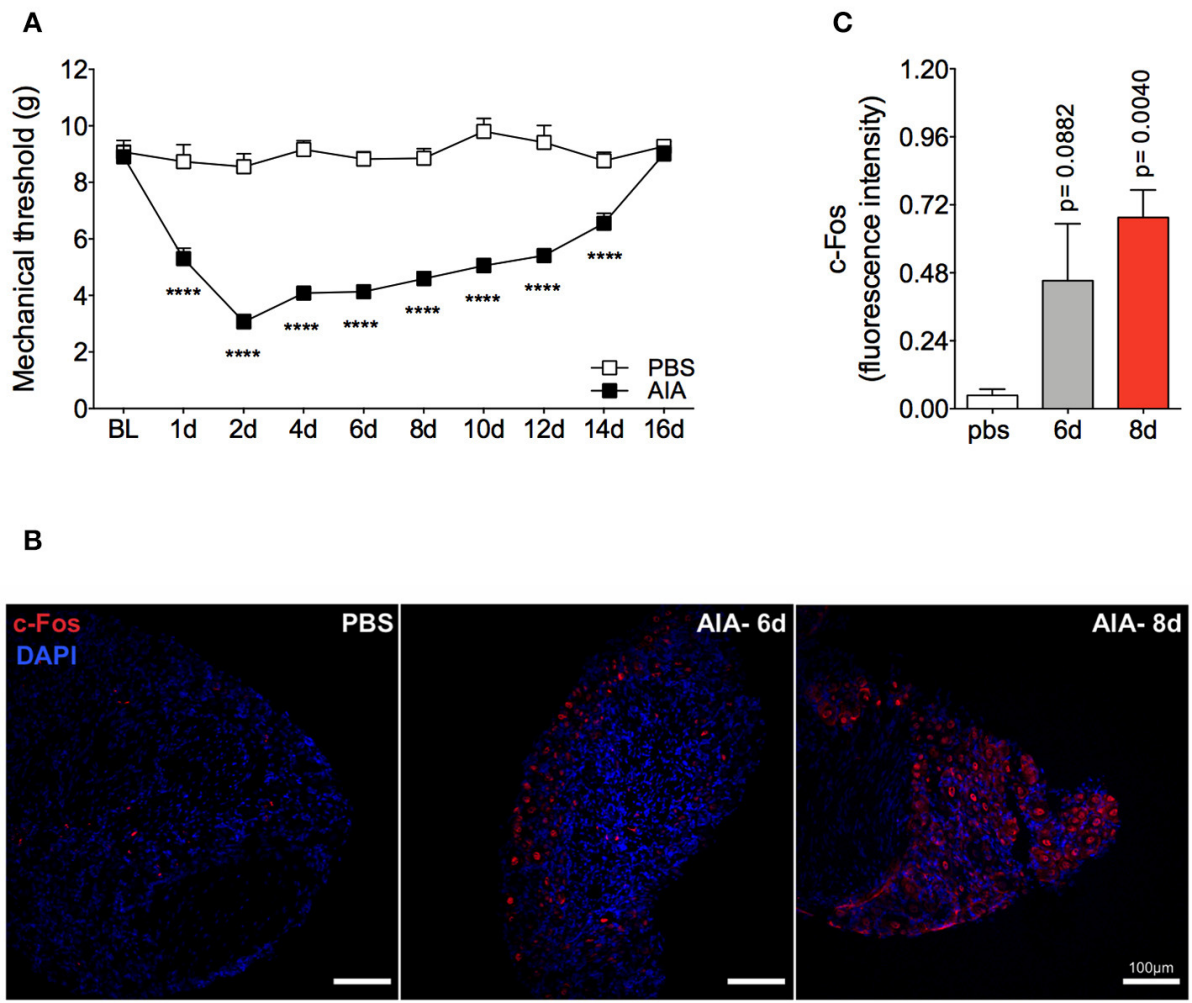
Persistent articular nociception in AIA model. Immunized mice received either i.a. injection of mBSA (10 μg/10 μl of PBS) to AIA induction or PBS (10 μl) in the control group. **(A)** Time course of nociceptive responses was recorded by electronic von Frey algesimeter. BL (baseline) (*n* = 6). **(B)** Confocal microscopy slides show c-Fos positive neurons within DRG (L4) 8 days after AIA induction and **(C)** its quantification. DRG samples from the control group were extracted 8 days after i.a. injection (*n* = 5–6). The results are represented as mean ± SEM; ****p < 0.0001 **(A)** or *p* = specified value in the graph **(C)** compared to control group (PBS) using two-way ANOVA following by Sidak *post-hoc* analysis **(A)** or by Dunnett *post-hoc* analysis **(C)**.

**Figure 4:**
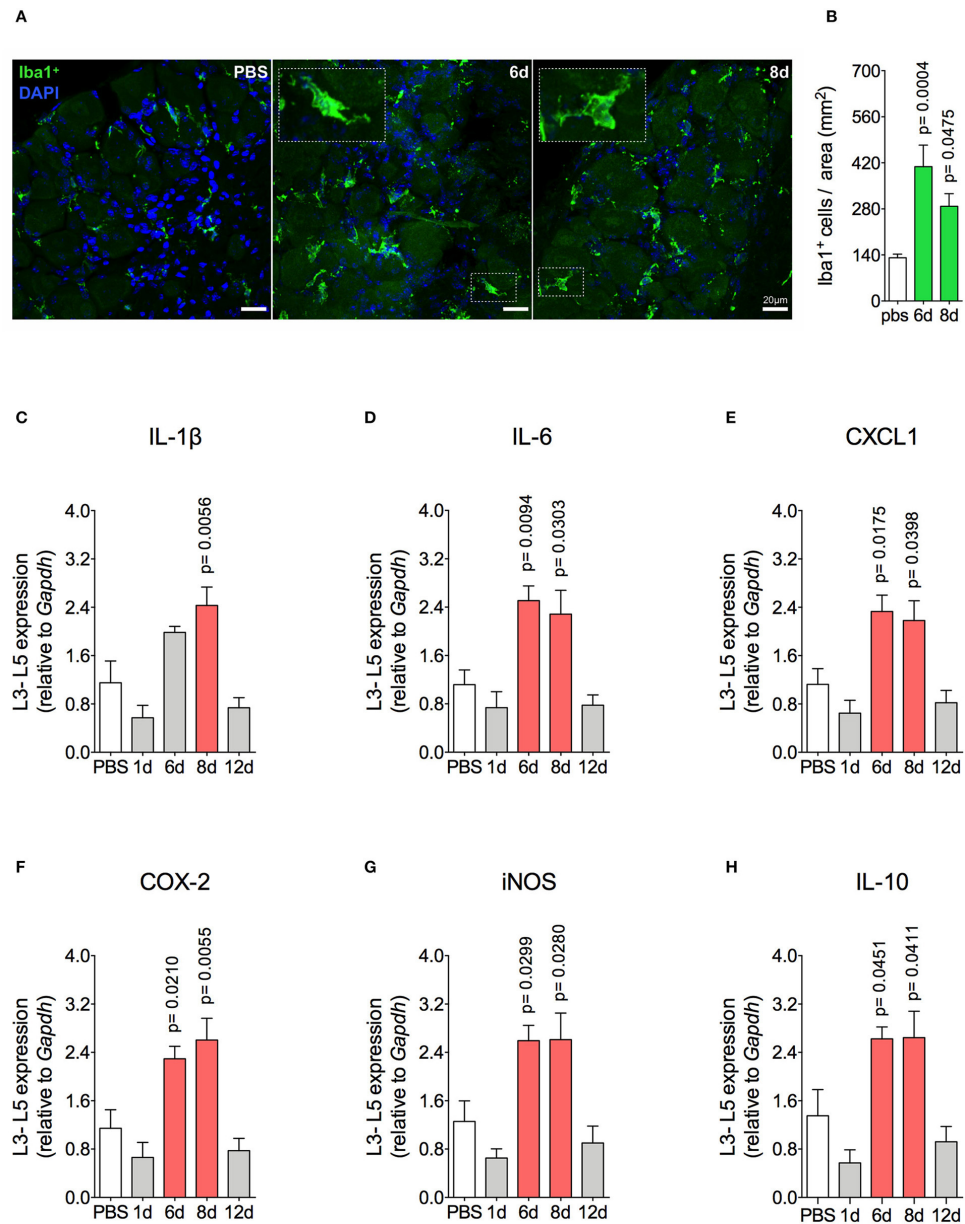
Resident macrophage activation and inflammatory molecules gene expression characterizes inflammatory status of DRG after resolution of inflammation in AIA model. Immunized mice received either i.a. injection of mBSA (10 μg/10 μl of PBS) to AIA induction or PBS (10 μl) in control group. **(A)** Confocal microscopy slides show marked Iba1^+^ cells within DRG (L4) 8 days after AIA induction and **(B)** its quantification (*n* = 5). Time course of gene expression of **(C)** IL-1β, **(D)** IL-6, **(E)** CXCL1, **(F)** COX-2, **(G)** iNOS, and **(H)** IL-10 within lumbar DRG (L3–L5). DRG samples from the control group were extracted 8 days after i.a. injection (*n* = 5). The results are represented as mean ± SEM; *p* = specified value in the graph compared to control group (PBS) using ANOVA following by Dunnett *post-hoc* analysis.

Mechanical joint nociception reached its peak at 2 days after joint challenge. However, although there was complete resolution of acute joint inflammation at day 8 post arthritis induction, mechanical joint nociception was persistent until day 14, returning to basal levels at day 16 (Figure 3A). In parallel, we confirmed the activation of the sensorial system in DRG by evaluating c-Fos expression after the inflammatory phase of AIA (Figures 3B,C). These findings suggest that sensory neuron excitability might be altered even after the resolution of acute joint inflammation. Several pain conditions can induce neuroinflammation in the sensory ganglion (21). DRG are the anatomical site where the cellular bodies of C and Aδ thin fibers, which sense nociceptive stimulus, are found. We observed an increase in Iba1^+^ cells (resident macrophage marker) in the DRG at 6 and 8 days (Figures 4A,B). This macrophage activation was accompanied by increased IL-1β gene expression at 8 days after AIA induction (Figure 4C). Moreover, expression of IL-6, CXCL1, COX2, and iNOS genes in the lumbar DRG (L3- L5) were also upregulated 6 and 8 days post joint challenge (Figures 4D–G). We also detected an increase in IL-10 gene expression at the same time points (Figure 4H). These results demonstrate that resolution of acute joint inflammation is restricted to the peripheral site in the first days after AIA induction and is followed by a transient inflammatory state in the sensory ganglia at later time points.

In summary, after resolution of acute inflammation in the challenged joint of immunized mice, there is an inflammatory environment in the DRG. This persistent local inflammatory state may be providing the nociceptive trigger factors that potentially sensitize neurons that maintain increased joint hyperalgesia following natural remission of AIA.

### Prolonged Joint Nociception Is Dependent on TNF in the Sensory Ganglia

**Figure 5:**
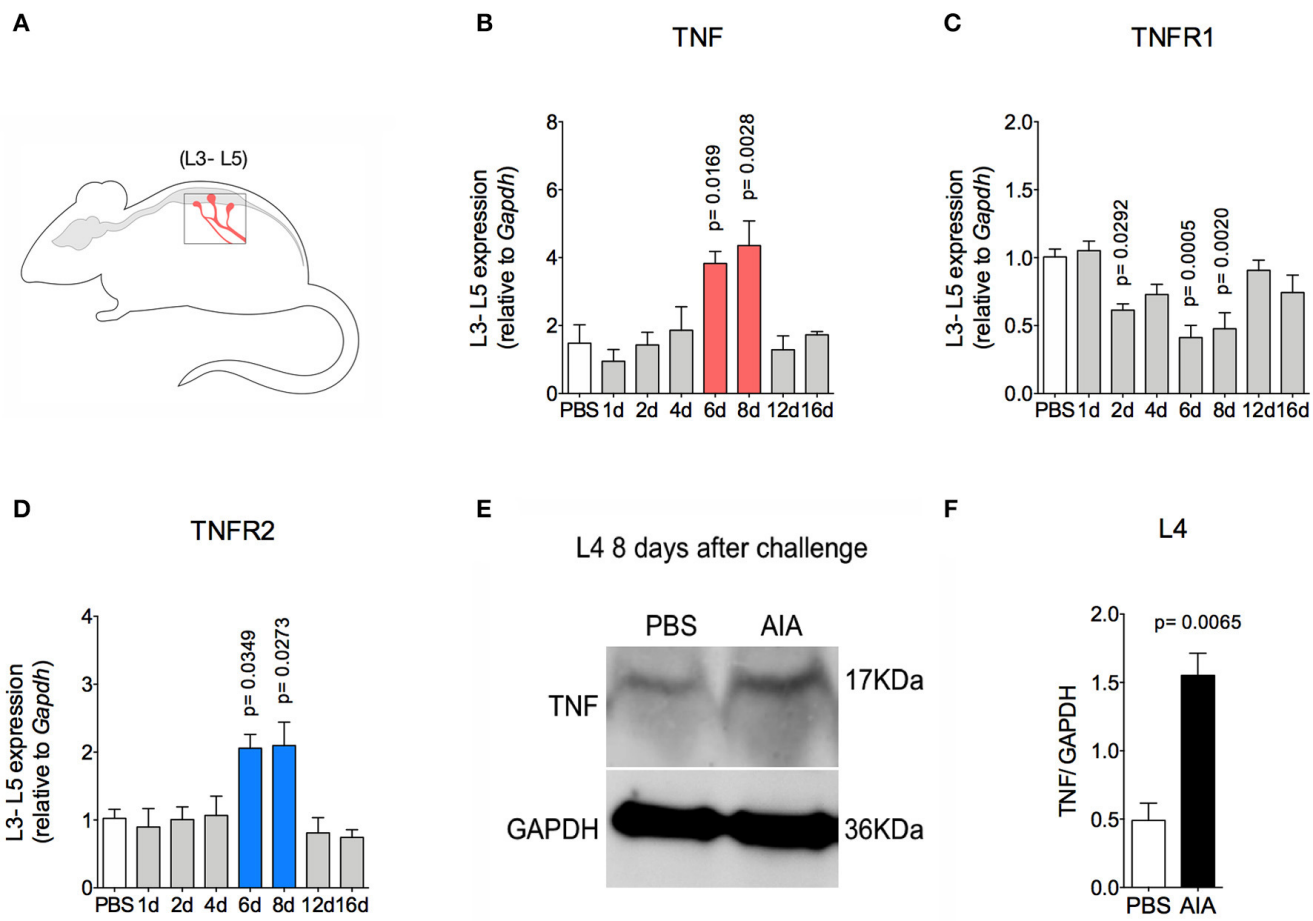
TNF expression within DRG after resolution of inflammation in AIA model. Immunized mice received either i.a. injection of mBSA (10 μg/10 μl of PBS) to AIA induction or PBS (10 μl) in control group. **(A)** Lumbar DRG (L3–L5) extracted at different time points after AIA (specified in the graphs **B–D**). **(B)** TNF and its receptors **(C)** TNFR1 and **(D)** TNFR2 gene expression evaluation (*n* = 5). **(E)** TNF expressed protein within DRG (L4) 8 days after AIA by western blot. **(F)** Densitometry referring to **(E)** (*n* = 3). DRG samples from the control group were extracted 8 days after i.a. injection. The results are represented as mean ± SEM; *p* = specified value in the graph compared to control group (PBS) using ANOVA following by Dunnett *post-hoc* **(B–D)** or two tailed unpaired *T*-test **(F)**.

TNF, a pro-nociceptive cytokine, is directly involved in various pain disorders, including joint pain in different forms of arthritis, due to central and peripheral actions (6, 27). However, without conditional peripheral inflammation, TNF has not been shown to elicit persistent pain in arthritis. Following the resolution of acute inflammation in the joint, there was an increase in TNF and TNFR2 gene expression 6 and 8 days after AIA induction in lumbar DRG (L3-5; Figures 5A,B,D). Moreover, increased TNF protein expression was detected in L4 DRG at day 8 (Figures 5E,F). However, this was not seen in the L3 or L5 DRG (data not shown). Additionally, there was no increase in TNFR1 gene expression (Figure 5C).

**Figure 6:**
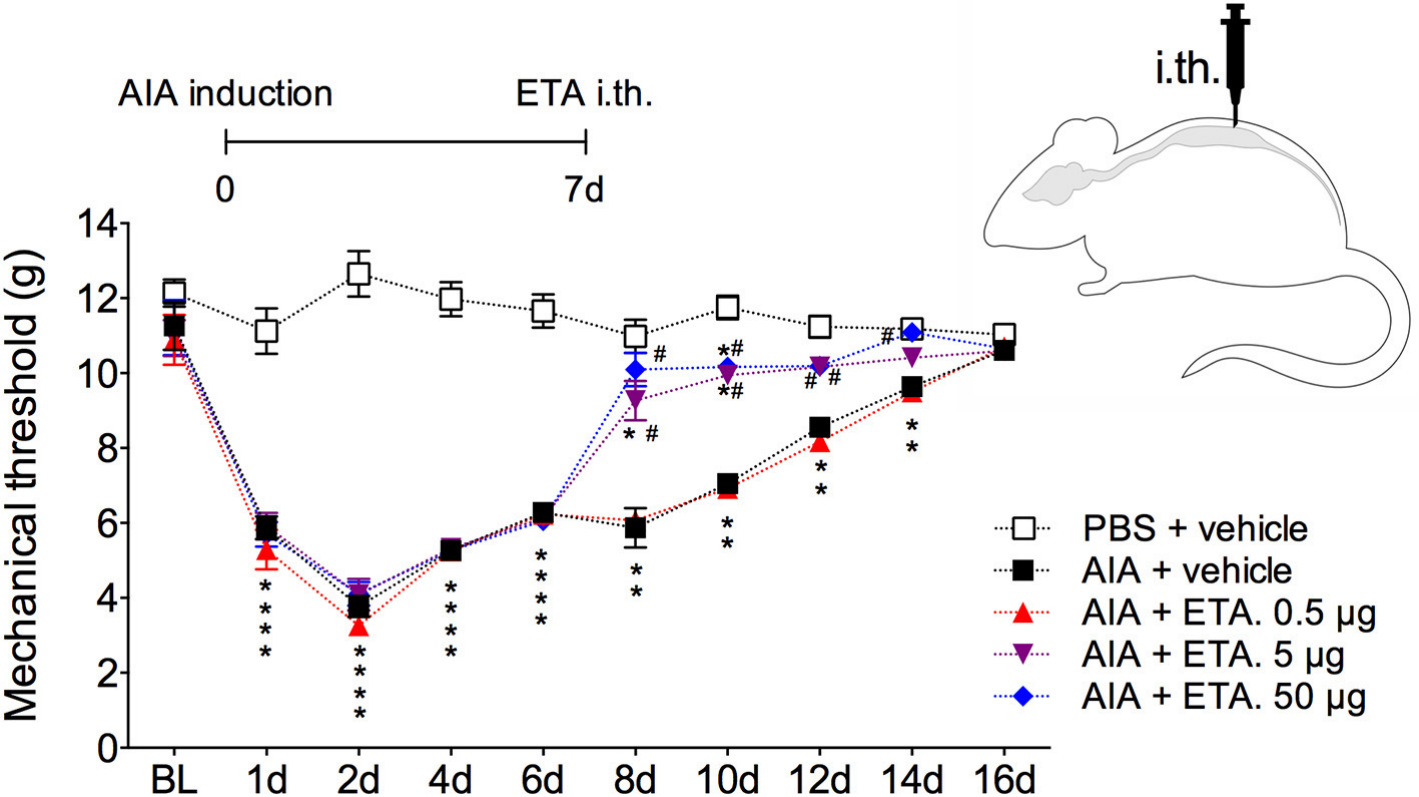
Persistent nociception is reversed after neutralization of TNF within DRG by intrathecal injection of etanercept. Immunized mice received either i.a. injection of mBSA (10 μg/10 μl of PBS) to AIA induction or PBS (10 μl) in control group. Time course of nociceptive response was recorded by electronic von Frey algesimeter. Etanercept (ETA; 0.5, 5, or 50 μg) was intrathecally (i.th.) administered on the 7th day after AIA. BL (baseline) (*n* = 7). The results are represented as mean ± SEM; *Compared to the control group (PBS) or ^#^compared to AIA + vehicle group when *p* < 0.05 using two-way ANOVA following by Dunnett *post-hoc* analysis.

**Figure 7:**
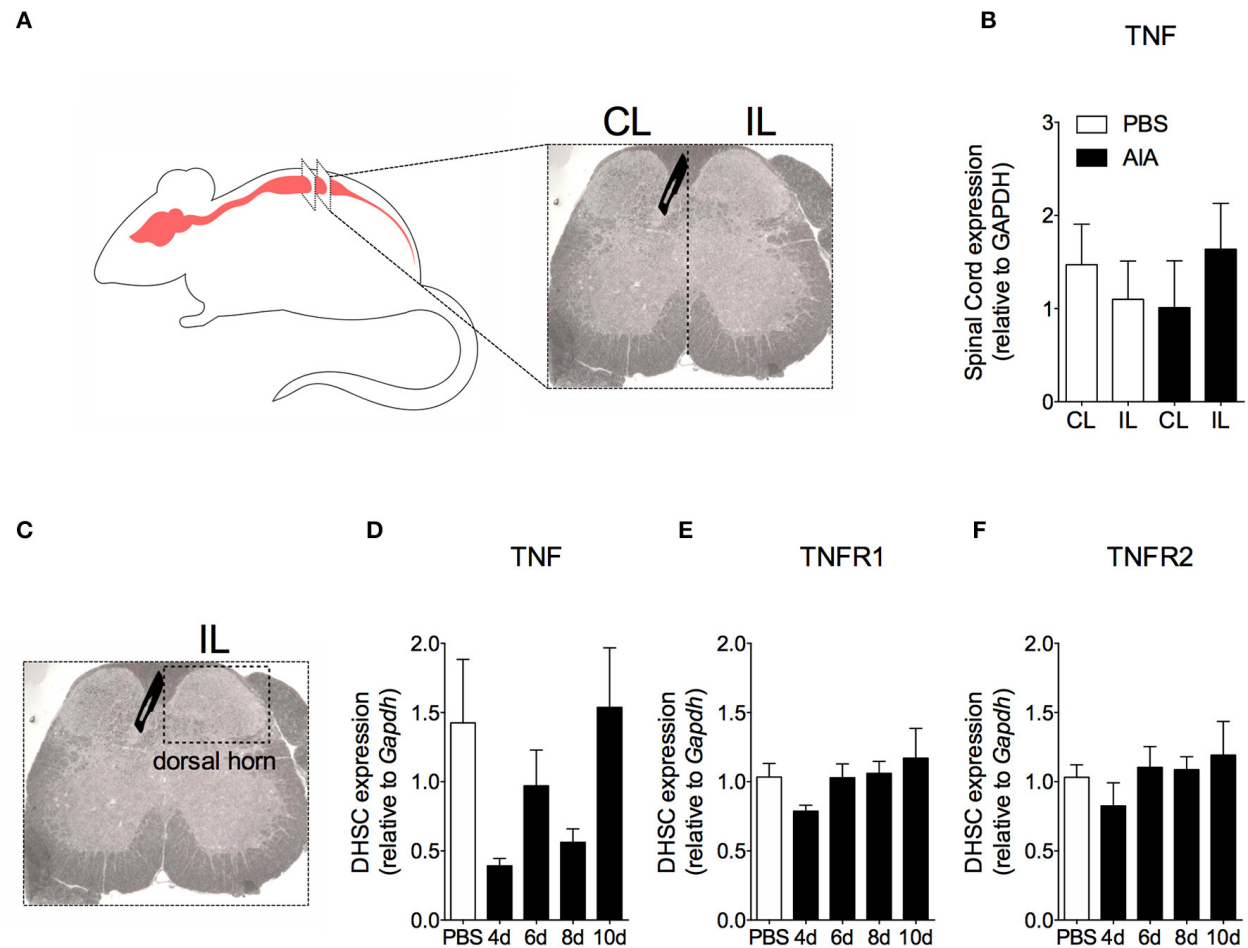
Time course of TNF and its receptors TNFR1 and TNFR2 gene expression within the spinal cord in AIA model. Immunized mice received either i.a. injection of mBSA (10 μg/10 μl of PBS) to AIA induction or PBS (10 μl) in control group. **(A)** Contralateral (CL) and ipsilateral (IL) lumbar intumescence. **(B)** Relative evaluation of TNF gene expression in CL and IL spinal cord of AIA and control mice 8 days after AIA induction (*n* = 5). **(C)** Dorsal horn of the spinal cord (DHSC). **(D)** TNF and its receptors **(E)** TNFR1 and **(F)** TNFR2 gene expression evaluation in the dorsal horn extracted at different time points (*n* = 8). Samples of the spinal cord of the control group were extracted 8 days after i.a. injection. The results are represented as mean ± SEM; (*n* = 5–8) to statistical analyses was used ANOVA following by Dunnett *post-hoc* analyses.

**Figure 8:**
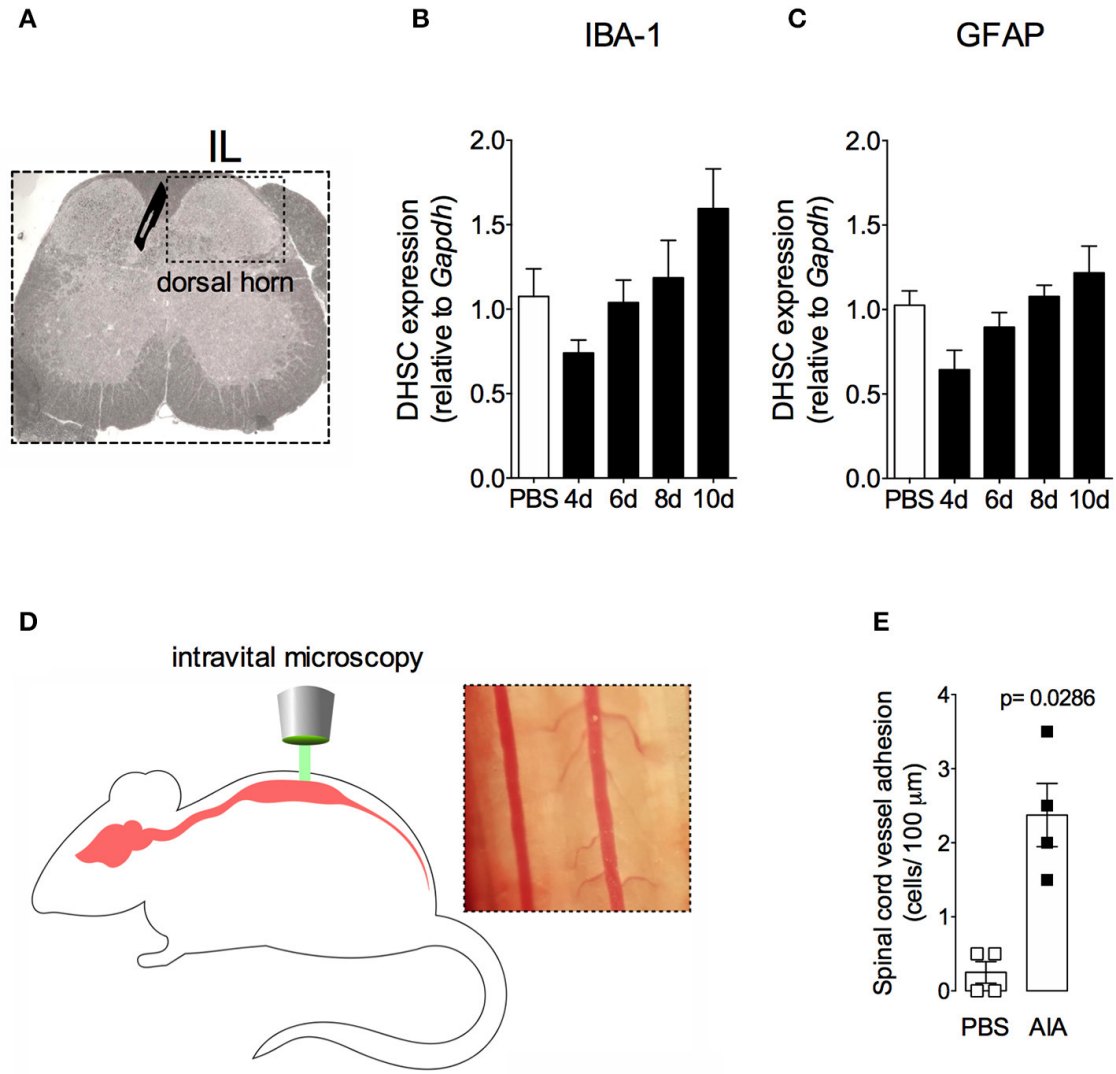
Non-reactive status of glial cells and leucocytes adhesion in the spinal cord after resolution of acute inflammation in AIA model. Immunized mice received either i.a. injection of mBSA (10 μg/10 μl of PBS) to AIA induction or PBS (10 μl) in control group. **(A)** Dorsal horn of the spinal cord (DHSC). **(B)** Iba1 and **(C)** GFAP relative evaluation of gene expression in the dorsal horn extracted at different time points (*n* = 8). Dorsal horn samples from the control group were extracted 8 days after i.a. injection. The results are represented as mean ± SEM; to statistical analyses was used ANOVA following by Dunnett *post-hoc* analyses. A laminectomy was performed 8 days after i.a. injection of mBSA or PBS to evaluate **(E)** adherent leucocytes (**D**, representative photo on right) in dorsal microvasculature of lumbar intumescence (**D**, schematic picture on left) by intravital microscopy (*n* = 4). The results are represented as mean ± SEM; (*n* = 8) *p* = specified value in the graph compared to control group (PBS) using two tailed unpaired *T*-test.

In order to demonstrate the active contribution of TNF expression in DRG for joint mechanical nociception, we neutralized TNF by intrathecal injection of etanercept at day 7 after AIA induction. This treatment permanently reversed the nociceptive response of AIA mice (Figure 6). Intrathecal injection allows injected substance to spread through the subarachnoid space to reach the DRG body-rich area (39, 40). However, this route also permits injected substances to access the spinal cord structures (e.g., the dorsal horn) (40). To eliminate the hypothesis that the anti-nociceptive effect of etanercept was due to its action in the surficial laminae of the dorsal horn of the spinal cord, we investigated TNF expression at this site. No changes were observed in TNF gene expression on the ipsilateral spinal cord (Figures 7A,B) or dorsal horn (Figures 7C,D) during the time points evaluated in this model. Additionally, neither TNFR1 nor TNFR2 gene expression were increased in the dorsal horn of the spinal cord (Figures 7E,F, respectively). In addition, there were no changes in IBA-1 and GFAP gene expression (Figures 8B,C), indicating normal activity of microglia and astrocytes within the dorsal horn of the spinal cord (Figure 8A). Although there were no changes in these two activating glial cells markers, there was slight vascular activation in the spinal cord. We observed an increase in leukocyte adhesion in the posterior region of lumbar intumescence by intravital microscopy (Figures 8D,E). The analyzed vessels are located near the dorsal roots of the sensitive ganglia that penetrate the dorsal horn of the spinal cord.

**Figure 9:**
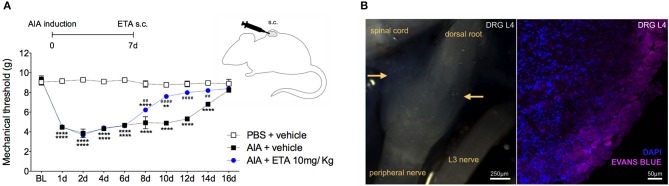
Articular hypernociception induced by TNF in the DRG is reversed after systemic injection of etanercept. Immunized mice received either i.a. injection of mBSA (10 μg/10 μl of PBS) to AIA induction or PBS (10 μl) in the control group. Etanercept (ETA; 10 mg/ Kg) was subcutaneously administered 7 days after AIA after the inflammatory phase in the joint. **(A)** Nociceptive response was recorded by electronic von Frey algesimeter. BL (baseline) (*n* = 5). **(B)** Arthritic mice received an intravenous injection of Evans Blue 8 days after AIA induction to DRG permeability evaluation. (**B**, left) Yellow rows indicate accumulation of Evans Blue around intact L4 DRG. (**B**, right) Confocal microscopy slide shows marked Evans Blue accumulated in body reach area of DRG (purple) and nuclei by DAPI (blue) within L4 DRG from AIA mice. The results are represented as mean ± SEM.; *Compared to control group (PBS) or ^#^compared to AIA + vehicle group when *p* < 0.05 using two-way ANOVA following by Tukey *post-hoc* analysis (a).

Therefore, we have demonstrated that TNF affects neutralization after intrathecal etanercept administration, specifically in the DRG but not in the nociceptive spinal cord regions. In a separate experiment, etanercept was given systemically 7 days after AIA induction. Similar to the intrathecal treatment, systemic neutralization of TNF also reversed joint nociception (Figure 9A). DRG have an extensive and fenestrated vasculature (41). To determine whether systemic anti-TNF treatment could reach the DRG structure, we evaluated vascular permeability in the DRG. Intravenous injection of Evans blue penetrated DRG, confirming its vascular permeability to high molecular weight molecules (Figure 9B). These results indicate a consistent possibility for systemic treatment with TNF inhibitors for pain relief even in the remission phase of joint inflammation due to the blockade of persistent TNF expression in the DRG.

## Discussion

Joint pain is a common and debilitating symptom in arthritic patients and different molecules produced in inflammatory environment directly activate peripheral sensory innervation, reducing the threshold for the nociceptor signal transduction. Using an acute model of arthritis in mice, we demonstrated here that joint nociception is detected for several days after complete resolution of acute joint inflammation and that acute neutrophilic inflammation in an AIA model was spontaneously resolved. In line with previous studies, the decreased number of neutrophils in our model is directly correlated to the resolving phase of joint inflammation and tissue repair (7). Here, there was also a decrease of cytokine production and improvement of tissue injury. These results highlighted that AIA presents an orchestrated series of events that eventually lead to self-limited resolution of inflammation (38). However, our results showed that nociception was persistent for several days after neutrophilic resolution in the challenged knee. Of note, this sensorial response was not triggered by peripheral action of sensitizing cytokines, TNF, IL-1β and IL-6, which are important mediators of the hyperalgesia evoked by AIA (6, 42–44). This persistent articular nociception was associated with continuous activation of non-neuronal cells and inflammatory status in the DRG, with crucial participation of TNF. The blockade of TNF with etanercept delivered by intrathecal or systemic routes was able to reverse the persistent nociception evoked by TNF in the DRG. It has been demonstrated that T cells may be involved in the transition from acute to chronic pain after inflammatory stimulus, indicating a role in pain maintenance of several inflammatory diseases (45). However, in our model, the number of mononuclear cells, which includes monocytes and lymphocytes, present after AIA did not differ from the basal level of cells from cavities injected with PBS. Thus, lymphocytes do not appear to be involved in pain maintenance in our model. Together, our findings suggest that delayed and continuous TNF production in the sensorial ganglia may drive persistent joint pain after the resolution of acute inflammation associated with arthritis.

There have been a number of studies which have tried to decipher neuro-immune interactions in the nociceptive apparatus and how that leads to increased pain sensitivity (21, 46). Changes in the DRG environment that explain persistent pain following arthritis remission are not fully understood. Hence, we had asked whether inflammatory stimuli in the DRG after the resolution of acute joint inflammation in AIA could be associated with persistent joint nociception. Our results show an increase in Iba1^+^ cells in DRG associated with elevated TNF, IL-1β, IL-6, COX2, and iNOS gene expression *in situ*. Our observations are consistent with the role of inflammatory molecules for the sensitization of neurons in DRG and the development of hyperalgesia (47–53). Interestingly, this neuroinflammation within the DRG was corroborated with the activation of leukocyte-endothelium interactions in the lumbar intumescence.

The presence of TNF in peripheral and central structures is fundamental for joint pain in arthritis (6, 14, 27, 44). During joint inflammation in experimental models of arthritis, up-regulated TNF in the spinal cord is implicated in increased articular nociception (27, 54). Quadros et al. (27) demonstrated that challenging the joints of immunized mice with a high dose of mBSA produce marked articular hypernociception that is associated with increased TNF expression and microglial activation in the spinal cord. These authors have also shown that TNF neutralization by intrathecal administration of infliximab, or spinal prevention of microglial activation by minocycline or fluorocitrate injection, ameliorates AIA-induced hypernociception. Similarly, reversion of persistent pain hypersensitivity in a model of collagen antigen-induced arthritis was induced by spinal inhibition of microglia and astrocyte activation after intrathecal minocycline and pentoxifylline administration, respectively (55). In contrast, we did not observe spinal overexpression of TNF, TNFR1, and TNFR2, nor did we observe overexpression of Iba1^+^ and GFAP^+^ cells, which are glial cell activation markers, during the persistent joint nociception. Therefore, we eliminated the hypothesis that etanercept could neutralize TNF produced in the spinal cord. However, intrathecal injection of etanercept after a complete resolution of acute joint inflammation was able to reverse persistent joint nociception. To explain our findings, we evaluated TNF expression in the DRG. We found increased TNF expression in the sensory ganglia after resolution of acute inflammation in the challenged joint. The action of TNF in the DRG is expected to produce a pro-nociceptive effect (21), and its overexpression in this sensorial organ is involved with pathological pain sensitization in models of neuropathic pain (56), acute herpetic neuralgia (57), and diabetes neuropathy (58). Thus, our work suggest that the source of TNF is Iba1^+^ macrophages within DRG 6 and 8 days post AIA. At these time points, we also observed both elevated TNF gene expression in lumbar ganglions (L3-L5) and increased amounts of TNF in L4 DRG. Our observations are consistent with a previous study showing that TNF expression in the DRG environment is produced by macrophages (47, 59). As a result, we suggested that articular hyperalgesia remaining after the resolving phase in AIA is triggered by overexpression of TNF, possibly from non-neuronal cells activated in the DRG.

Within the DRG, inflammatory conditions also enhances TNFR1 expression in either non-neuronal cells and nociceptors, while increased TNFR2 expression is restricted in non-neuronal cells (59). In arthritis, the profile of changes in TNF receptors in DRG is not clear in the literature. In a model of arthritis induced by complete Freund's adjuvant, Inglis et al. (30) had demonstrated that both TNFR1 and TNFR2 expression were increased in neurons and infiltered macrophages into the DRG, respectively. However, Boettger et al. (28) showed expression of both receptors on small- and medium-sized DRG neurons, but the proportion of TNFR1 and TNFR2 positives cells did not alter the course of AIA. Here, TNFR1 gene expression in DRG was decreased after the resolving phase 6 and 8 days after AIA. On the other hand, increased TNFR2 gene expression was observed in DRG 8 days after AIA induction. In fact, macrophages infiltrating the DRG during arthritis express TNFR2 and are associated with articular pain (30, 31). In addition, these TNFR2 expressing immune cells could be a second pathway to neuronal modulation by TNF (30). Since local and systemic neutralization of TNF by etanercept decreased the persistent articular hypernociception, our data suggest that expression of both TNF and TNFR2 in DRG might be necessary to maintain hypernociception. Of note, TNF produced peripherally contributes to inflammation and sensitizes nociceptive endings in the joints structures (6, 13). Therefore, systemically delivered etanercept could target articular tissue. We also evaluated the antinociceptive effect of this drug when TNF levels returned to basal level in articular sites. Thus, we eliminated the hypothesis that decreased nociceptive response after systemic administration of etanercept could be associated with articular TNF neutralization. Taken together, systemic administration of etanercept may be beneficial against clinical manifestation of both articular inflammation and pain. These perspective highlight the potential advantages for systemic drug administration in clinician practice, that includes easy application and widespread actions of injected agents (39). In order to improve the clinical translation, the use of humanized chimeric mouse models needs to be explored.

In conclusion, we have demonstrated that natural resolution may not contribute to pain recovery once TNF remains active in the DRG. However, whether exogenous treatment with pro-resolving mediators decrease the levels of TNF in the DRG and alleviate pain still needs to be clarified. Our data show that TNF targeting DRG represents a mechanism independent of spinal sensitization which elicits articular hypernociception after resolution of acute joint inflammation.

## Data Availability Statement

The raw data supporting the conclusions of this article will be made available by the authors, without undue reservation, to any qualified researcher.

## Ethics Statement

The animal study was reviewed and approved by the Animal ethics committee of Federal University of Minas Gerais.

## Author Contributions

WG, TC, MT, FA, and VP designed the study, performed the data analysis, and wrote the manuscript. WG prepared all figures. WG, BR, MO, LR, VF, WS, PP, CQ-J, KS, WC, VB, VC, AB, WV, and FL conducted the experiments and reviewed the manuscript.

### Conflict of Interest

The authors declare that the research was conducted in the absence of any commercial or financial relationships that could be construed as a potential conflict of interest.
